# Impact of Selected Pre-Analytical and Analytical Factors on Untargeted Salivary Metabolomics

**DOI:** 10.3390/ijms27083345

**Published:** 2026-04-08

**Authors:** Sylwia Michorowska, Agnieszka Zięba, Dorota Olczak-Kowalczyk, Joanna Giebułtowicz

**Affiliations:** 1 Department of Drug Chemistry, Pharmaceutical and Biomedical Analysis, Medical University of Warsaw, 02-091 Warszawa, Poland; sylwia.solobodowska@wum.edu.pl (S.M.); zieba.agnieszka@hotmail.com (A.Z.); 2 Department of Pediatric Dentistry, Medical University of Warsaw, 02-091 Warszawa, Poland; dorota.olczak-kowalczyk@wum.edu.pl

**Keywords:** mass spectrometry, method development, pre-analytical factors, saliva, untargeted metabolomics

## Abstract

With the growing interest in personalized medicine, alternative biological matrices to blood are increasingly explored as sources of diagnostic information. Saliva has emerged as a promising diagnostic matrix due to its non-invasive collection, suitability for home sampling, and minimal requirements for personnel training. Numerous studies have demonstrated the presence of metabolites in saliva that enable disease diagnosis and monitoring. However, the influence of pre-analytical and analytical factors on salivary metabolomics outcomes remains insufficiently characterized. In this study, we investigated factors potentially affecting the number and abundance of detected metabolites in untargeted salivary metabolomics using liquid chromatography coupled with mass spectrometry (LC–MS). The impact of chromatographic column type, extraction protocol, and saliva type (stimulated versus resting) was evaluated. Additionally, the effect of swab type on analyte recovery was assessed. The use of a synthetic swab for saliva collection yielded results most comparable to those obtained without swabs, for both resting and stimulated saliva samples, indicating minimal pre-analytical interference. The greatest metabolite coverage was obtained using ACN:MeOH (1:1, *v*/*v*), with a ZIC-HILIC column for polar metabolites and a C18 column for non-polar metabolite separation. These findings demonstrate that swab type, chromatographic column, extraction solvent, and saliva type critically shape metabolite coverage in untargeted salivary metabolomics. Importantly, the distinct metabolic profiles of resting and stimulated saliva suggest that these matrices may provide complementary clinical insights, underscoring the need for saliva type selection tailored to specific diagnostic and biomarker discovery objectives.

## 1. Introduction

The advancement of personalized medicine has redefined diagnostic and therapeutic approaches by integrating patient-specific biological profiles into clinical decision-making. This shift has been accelerated by progress in genomics, systems biology, and high-throughput data analytics, alongside the proven efficacy of targeted therapies with minimized adverse effects. Moreover, substantial interindividual variability in treatment response has highlighted the necessity for tailored approaches in managing complex diseases [1].

As the global healthcare system faces mounting economic pressures, workforce shortages, and the demand for frequent monitoring, there is an increasing need for minimally invasive, cost-effective alternatives to conventional blood-based diagnostics. Saliva has emerged as a compelling candidate in this context. Easily accessible, non-invasive, and inexpensive to collect and process, saliva reflects systemic physiological and pathological states, making it a valuable biofluid for clinical and translational research [2].

Metabolomics—defined as the comprehensive analysis of low-molecular-weight metabolites—has become a key tool in systems biology, enabling detailed characterization of cellular responses, disease mechanisms, and therapeutic effects. The application of metabolomic profiling to saliva provides a unique opportunity to access biomarker-rich information non-invasively, with potential utility in early disease detection, patient stratification, and real-time health monitoring [3].

Despite the complexity of factors affecting salivary metabolite composition, interest in saliva as a diagnostic biofluid continues to grow. Several saliva-based assays have already been implemented to quantify clinically relevant analytes such as cortisol, estradiol, progesterone, and testosterone [4]. Salivary cortisol is routinely used in the diagnosis of Cushing’s syndrome and is currently being investigated for the identification of patients with adrenal insufficiency [5]. Saliva-based measurements of testosterone and estradiol support the evaluation of androgenic disorders and ovulatory dysfunctions, respectively [6], while melatonin levels serve as circadian biomarkers in sleep–wake disorder diagnostics [7]. Additionally, saliva is increasingly applied in therapeutic drug monitoring, highlighting its clinical utility as a minimally invasive matrix [8].

While salivary metabolomics has seen growing interest in recent years, significant analytical challenges remain. Compared to plasma, the salivary matrix is less studied, and limited data are available on pre-analytical and analytical variables affecting metabolite extraction, stability, and detection [4,9,10,11]. Most prior studies have focused on a narrow range of extraction solvents, typically acetonitrile or acetonitrile/methanol mixtures [12,13], with limited comparative evaluation of alternatives such as isopropanol or methyl tert-butyl ether, which are frequently employed for the extraction of hydrophobic metabolites from other biofluids. Similarly, there is a lack of systematic comparison across chromatographic separation columns for salivary metabolomics, despite extensive research emphasizing the importance of column selection in plasma-based studies. For instance, Boudah et al. systematically evaluated the performance of C18, ZIC-HILIC, and HS F5-PFPP columns for untargeted metabolomics of plasma samples, demonstrating substantial differences in metabolite coverage and separation efficiency [10]. However, no equivalent comparative studies have been conducted using saliva as the analytical matrix.

Another unresolved question is the impact of saliva type (resting versus stimulated) on the metabolic fingerprint obtained through untargeted metabolomics using liquid chromatography–tandem mass spectrometry (LC-MS/MS) analysis. Despite the widespread use of both types of saliva, few studies have directly compared their analytical performance in metabolomic workflows [14,15,16]. To date, only one study has employed LC-MS/MS for such a comparison [17]. Moreover, no studies have compared the impact of using cotton versus synthetic swabs for saliva collection on salivary metabolite recovery.

To address these gaps, the present study systematically investigates the influence of extraction protocol, chromatographic column selection, and saliva and swab type on the performance of untargeted salivary metabolomics using LC-MS/MS (Figure S1). The findings aim to support the development of standardized protocols for saliva-based diagnostics and improve the reliability of salivary metabolomic data in clinical and research applications.

## 2. Results

### 2.1. Selection of the Extraction Protocol

**Figure 1:**
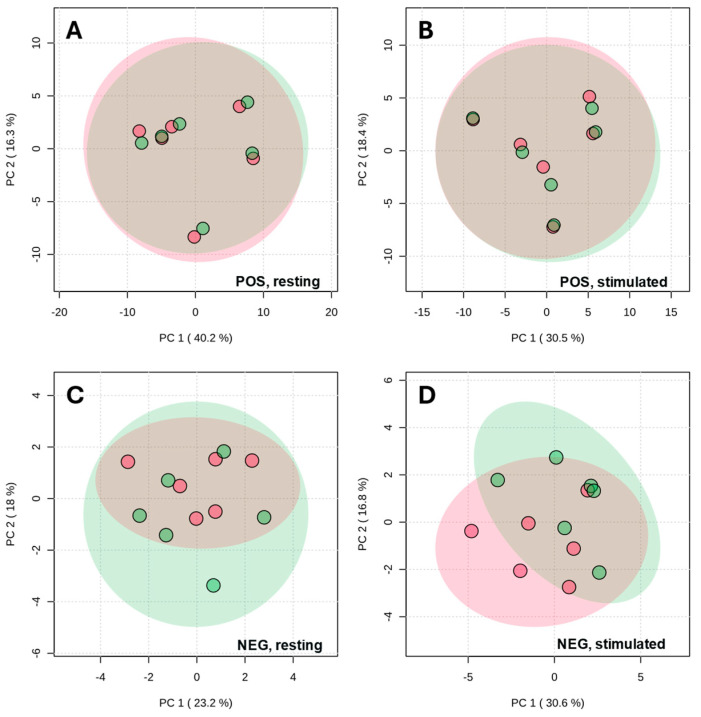
Principal component analysis (PCA) of metabolic profiles of stimulated and resting saliva, with metabolites separated in a ZIC-HILIC column and detected in both positive (POS) and negative (NEG) ionization modes, following extraction with acetonitrile (ACN, green) and an acetonitrile/methanol mixture (ACN:MeOH (1:1, *v*/*v*), red). (**A**) Resting saliva measured in positive mode; (**B**) stimulated saliva measured in positive mode; (**C**) resting saliva measured in negative mode; (**D**) stimulated saliva measured in negative mode.

As an initial step, extraction protocols were systematically compared. Two procedures were evaluated with chromatographic separation performed using a ZIC-HILIC column: extraction with acetonitrile (ACN) and with an acetonitrile/methanol mixture (ACN:MeOH (1:1, *v*/*v*)). Principal component analysis (PCA), which reduces complex datasets to highlight similarities and differences between samples, score plots (Figure 1) of metabolites separated on the ZIC-HILIC column demonstrated a high degree of overlap between the two extraction protocols across both ionization modes and for both stimulated and resting saliva (Figure 1). This indicates that both extraction methods yielded similar broadly comparable metabolic coverage. Minor differences were observed in negative ionization mode, particularly for stimulated saliva, suggesting a limited extraction-dependent effect on a subset of metabolites. 

**Figure 2:**
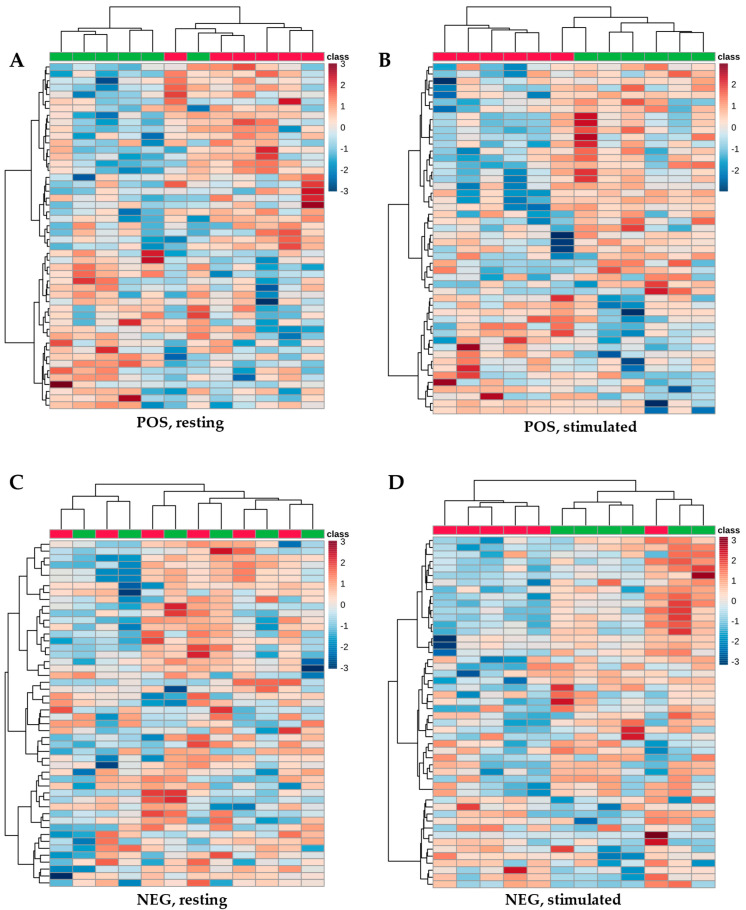
A heatmap of the 50 most discriminating metabolites (lowest *p*-values using *t*-Student’s test) separated on the ZIC-HILIC column. Red indicates metabolite abundances above the mean, whereas blue denotes abundances below the mean. Red corresponds to extraction with acetonitrile/methanol (ACN:MeOH (1:1, *v*/*v*)), and green to extraction with acetonitrile (ACN). (**A**) Resting saliva measured in positive mode (POS); (**B**) stimulated saliva measured in positive mode (POS); (**C**) resting saliva measured in negative mode (NEG); (**D**) stimulated saliva measured in negative mode (NEG).

Heatmaps were generated to further visualize differences in metabolite abundances across extraction protocols. For each comparison, the 50 most discriminating metabolites were displayed. Red indicates metabolite abundances above the mean, whereas blue denotes abundances below the mean. The heatmaps were consistent with the PCA results (Figure 2). No pronounced differences were detected between extraction protocols, irrespective of ionization mode or saliva type, indicating similar metabolite abundances.

The trends observed in the chemometric analyses were corroborated by classical statis-tical tests, which revealed no significant differences in metabolite abundances between ACN- and ACN:MeOH-based extractions (all changes with *p* > 0.05).

**Figure 3:**
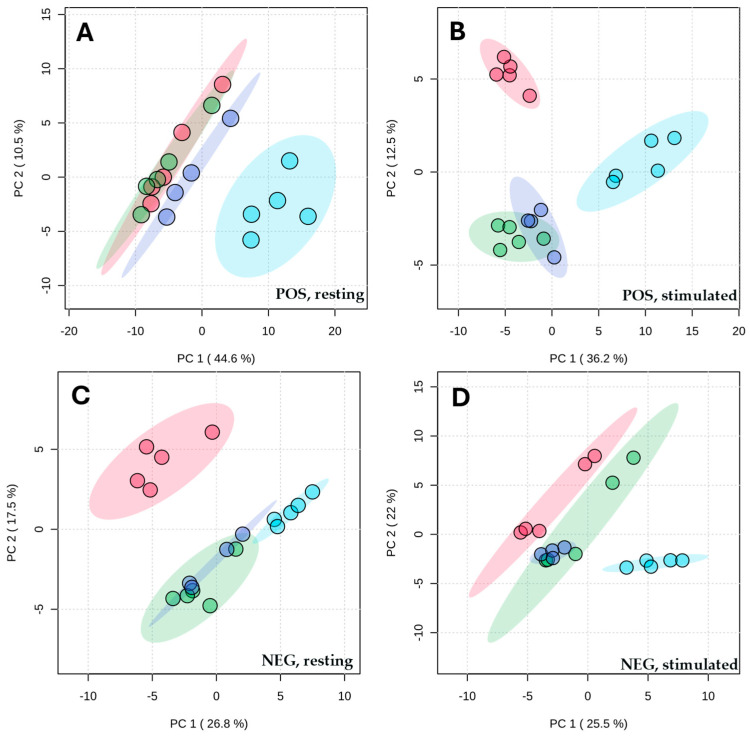
Principal component analysis of metabolic profiles of stimulated and resting saliva, with metabolites separated on a C18 column and detected in both positive (POS) and negative (NEG) ionization modes, following extraction with acetonitrile (ACN, green), an acetonitrile/methanol mixture (ACN:MeOH (1:1, *v*/*v*), red), isopropanol (IPA (violet)), or methyl tert-butyl ether (MTBE (blue)). (**A**) Resting saliva measured in positive mode; (**B**) stimulated saliva measured in positive mode; (**C**) resting saliva measured in negative mode; (**D**) stimulated saliva measured in negative mode.

For metabolites separated on a reversed-phase column, four extraction protocols were compared: ACN, ACN:MeOH (1:1, *v*/*v*), IPA, and MTBE. MTBE-based extraction yielded a metabolic profile that differed most from those obtained with the other protocols. In negative ionization mode, metabolites extracted with ACN:MeOH (1:1, *v*/*v*) also clearly differed from those extracted with both ACN and IPA, whereas extractions performed with ACN and IPA yielded highly similar profiles (Figure 3). 

**Figure 4:**
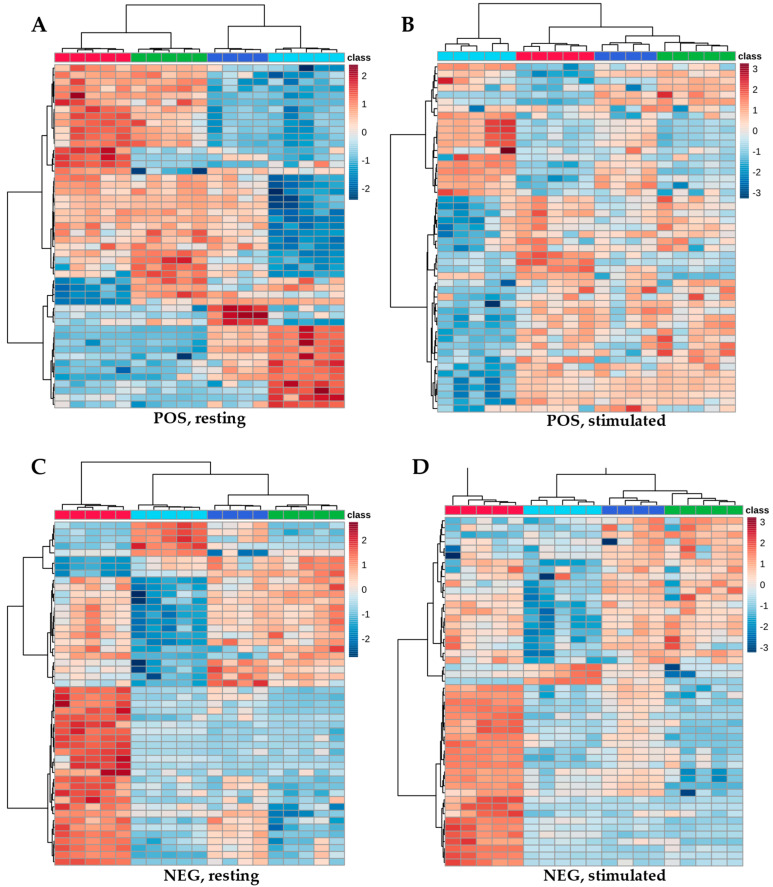
A heatmap of the 50 most discriminative metabolites (highest *p*-values using ANOVA test) separated on the C18 column in both positive (POS) and negative (NEG) ionization modes, following extraction with ACN (green), ACN:MeOH (red), IPA (dark blue), or MTBE (blue). Red indicates metabolite abundances above the mean, whereas blue denotes abundances below the mean. PD denotes resting saliva, and S denotes stimulated saliva. (**A**) Resting saliva measured in positive mode; (**B**) stimulated saliva measured in positive mode; (**C**) resting saliva measured in negative mode; (**D**) stimulated saliva measured in negative mode.

Heatmap analysis revealed the highest metabolite abundances in samples extracted with ACN:MeOH (1:1, *v*/*v*), whereas significantly lower abundances were observed following the MTBE-based extraction protocol (Figure 4). No significant differences were observed among the other extraction solvents. 

To facilitate interpretation of the differences between extraction solvents, the discriminating metabolites were grouped by chemical superclasses. For stimulated saliva (Figure S2), the MTBE-based extraction protocol yielded lower abundances across all metabolite superclasses compared with ACN:MeOH. The ACN protocol produced a metabolite profile largely similar to that obtained with ACN:MeOH. In contrast, the IPA-based procedure resulted in reduced abundances of lipid species and organic acids and derivatives, while other superclasses showed levels similar to those observed for ACN:MeOH extraction. For resting saliva (Figure S3), the MTBE protocol again yielded lower abundances of all metabolites compared with ACN:MeOH. Both ACN and IPA extraction protocols provided overall superclass distributions similar to that obtained with ACN:MeOH.

### 2.2. Selection of the Column

To select the optimal chromatographic separation conditions, we compared the number of features detected by MS/MS analysis following ACN:MeOH extraction and chromatographic separation. Among the reversed-phase columns (C18 and PFP) dedicated to the separation of non-polar compounds, a higher total number of features (calculated as the sum of those detected in both stimulated and resting saliva) was observed using the C18 column, with 571 features detected in positive ionization mode and 327 in negative ionization mode, compared with the PFP column, which yielded 508 and 249 features in positive and negative ionization modes, respectively. For the separation of polar metabolites, the ZIC-HILIC column led to the detection of a greater total number of features in positive ionization mode (630 vs. 421), whereas the amide HILIC column yielded more features in negative ionization mode (280 vs. 151).

### 2.3. The Influence of Saliva Type

**Figure 5:**
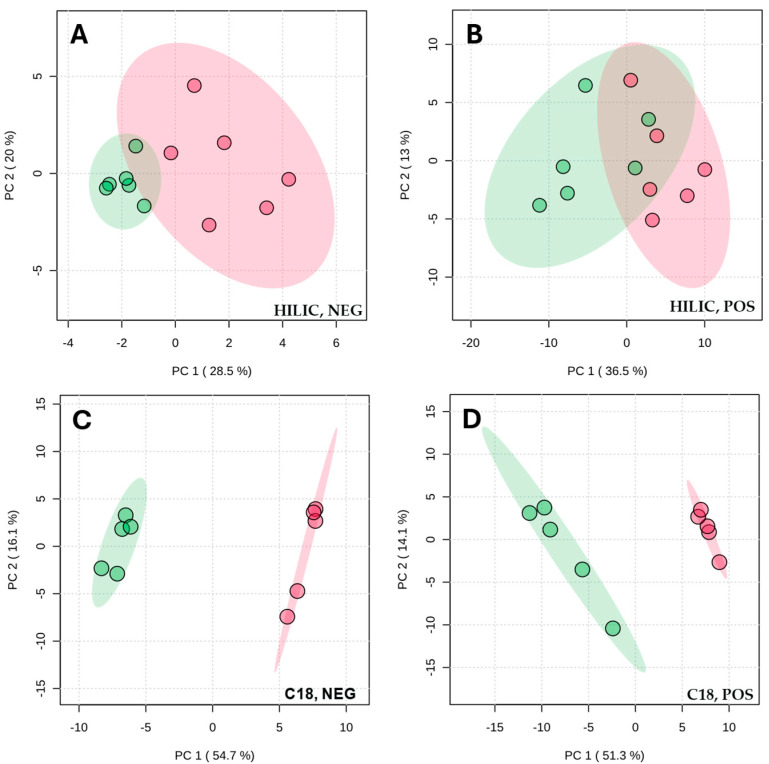
Principal component analysis of metabolic profiles of stimulated (red) and resting saliva (green), with metabolites separated on a C18 and HILIC columns and detected in both positive (POS) and negative (NEG) ionization modes, following extraction with an acetonitrile/methanol mixture (ACN:MeOH (1:1, *v*/*v*)). (**A**) HILIC, negative mode; (**B**) HILIC, positive mode; (**C**) C18, negative mode; (**D**) C18, positive mode.

**Figure 6:**
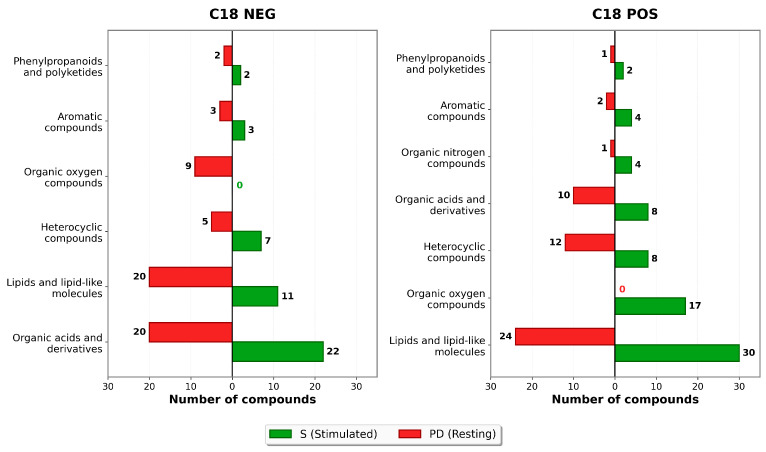
The distribution of metabolite superclasses detected after separation on the C18 column following acetonitrile/methanol (ACN:MeOH (1:1, *v*/*v*)) extraction in positive (POS) and negative (NEG) ionisation mode. The bar plot compares stimulated and resting saliva samples. Green bars represent metabolites from a given superclass that are more abundant in stimulated saliva, with the number indicating their count. Red bars represent metabolites from the same superclass that are more abundant in resting saliva, with the number indicating their count.

In the next step, stimulated and resting saliva were compared. Using PCA, which re-duces complex datasets to highlight similarities and differences between the samples, we observed partial overlap of clusters representing stimulated and resting saliva in both positive and negative ionization modes after separation on the ZIC-HILIC column (Figure 5). In contrast, PCA performed using data collected with the C18 column revealed clear and complete separation of stimulated and resting saliva samples in each ionization mode, indicating significant differences in terms of the metabolites detected in these two saliva types. Subsequently, discriminating metabolites separated on the C18 column were grouped into metabolite superclasses. Only superclasses showing differences in peak area for at least two metabolites between stimulated and resting saliva were included. Figure 6 illustrates the obtained results, with bars colored by saliva type: green for metabolites more abundant in stimulated saliva and red for those more abundant in resting saliva. The numbers indicate the count of metabolites in each category.

In negative ionization mode, more metabolites belonging to organic acids and their derivatives, as well as heterocyclic compounds, had higher abundance in stimulated saliva. In contrast, resting saliva showed higher numbers of more abundant lipids, lipid-like molecules, and oxygen-containing organic compounds. In positive ionization mode, stimulated saliva was characterized by more lipids and lipid-like molecules, oxygen-containing organic compounds, nitrogen-containing organic compounds, phenylpropanoids, and polyketides, whereas resting saliva exhibited a greater number of more abundant heterocyclic compounds and organic acids and their derivatives.

Taken together, the results obtained in both ionization modes after separation of metabolites on the C18 column indicate that the choice between stimulated and resting saliva should be tailored to the metabolite classes of interest. For example, stimulated saliva appears better suited for the analysis of organic nitrogen compounds.

### 2.4. The Influence of Swab Type

Finally, we examined how incubating resting and stimulated saliva with commonly used collection swabs affected metabolite abundances, determined using the LC-MS/MS platform. For resting saliva, the synthetic swab affected the measured abundance of 149 metabolites separated on the HILIC column and 58 metabolites separated using the C18 column. Incubation with the cotton swab changed the measured abundance of 215 and 143 metabolites separated on the HILIC and C18 columns, respectively. For stimulated saliva, incubation with the synthetic swab affected the measured abundance of 136 (HILIC column) and 109 (C18 column) metabolites, whereas the cotton swab altered 224 and 84 metabolites, respectively, (Figure S4). Although the use of swabs improved reproducibility, it also altered LC-MS/MS metabolite profiles. The synthetic swab affected saliva composition and subsequent measurement less and yielded metabolic profiles most similar to those of the control samples. It was therefore selected for the collection of both resting and stimulated saliva.

## 3. Discussion

To maximize the biological and clinical information obtained from salivary metabolomics, it is essential to understand how methodological factors influence both metabolite detection and quantification. Previous studies have shown that chromatographic column chemistry, extraction solvent, and saliva type are key determinants of metabolomic readouts [17,18]. Accordingly, in this study, we provide the first comprehensive evaluation of four chromatographic columns (ZIC-HILIC, amide HILIC, C18, and PFP) and four extraction protocols (ACN, ACN:MeOH (1:1, *v*/*v*), IPA, and MTBA) in a single systematic comparison, while also examining the influence of saliva type and collection method. This approach allowed us to clarify how each analytical choice—solvent, column, saliva and swab type—shapes metabolite coverage determined by LC-MS/MS, providing critical insights to guide standardized, reliable workflows for untargeted salivary metabolomics.

We first evaluated the impact of extraction protocol. ACN:MeOH (1:1, *v*/*v*) provided metabolite coverage comparable to that obtained with ACN alone. However, previous targeted studies have reported a lower matrix effect for ACN:MeOH compared with ACN, an observation also confirmed in plasma by Lepoittevin et al. [11]. Based on these findings, ACN:MeOH (1:1, *v*/*v*) was selected for subsequent analyses. The use of a 1:1 (*v*/*v*) ACN:MeOH mixture has also been reported by Chen et al. [19] and DeFelice et al. [12]. Most previous salivary metabolomics studies have assessed only one or two extraction solvents, most commonly ACN [13,20], MeOH [21,22], or their mixtures [23,24]. In our study, the most pronounced differences in both metabolite class distribution and abundance were observed between extraction protocols with ACN:MeOH and MTBE. IPA extraction resulted in reduced abundances of selected metabolite groups, particularly lipids and organic acids, compared with ACN and ACN: MeOH extractions. Although IPA is frequently used for plasma extraction [25], its performance in saliva was suboptimal under the conditions applied in this study.

Next, we compared chromatographic columns. Two columns dedicated to hydrophobic compound separation (C18 and PFP) and two columns optimized for hydrophilic compounds (ZIC-HILIC and amide HILIC) were evaluated. Our results demonstrate that the combined use of C18 and ZIC-HILIC columns provides the most comprehensive coverage of the salivary metabolome. Direct comparison with previous studies is limited, as most reports employed only two complementary chromatographic columns without clearly stated selection criteria. For example, Liu et al. [23] used HILIC Acquity UPLC BEH and RPLC Acquity UPLC HSS columns, while Martias et al. [24] applied C18 and HILIC Waters columns. Nevertheless, our findings are consistent with the broader literature, in which C18 columns are most frequently used for the separation of hydrophobic salivary metabolites, as reported by Assad et al. [26] and Schulte et al. [27], whereas ZIC-HILIC columns are commonly applied for the metabolomic analysis of polar compounds, as demonstrated by Teruya et al. [28] and Chen et al. [19].

Here, we also evaluated whether incubation of saliva with collection swabs affects metabolite abundances determined by using the LC-MS/MS platform. We demonstrated that incubation with synthetic swabs produced results most comparable to control samples incubated without any swab, indicating minimal pre-analytical interference. This is particularly relevant given that contact with sorbent materials is a known source of bias in salivary metabolomics [29]. Passive drooling or spitting is widely regarded as the preferred method of saliva collection for untargeted metabolomics, as it provides broad metabolite coverage while avoiding interactions with sorbents. However, these approaches may unintentionally stimulate salivary flow due to participant-induced oral activity aimed at accelerating saliva secretion, resulting in partial conversion from resting to stimulated saliva and compromised standardization. Swab-based devices (e.g., Salivette^®^, SalivaBio, Salimetrics) are therefore commonly used for their hygiene and ease of handling [30], despite limited evidence regarding their analytical suitability.

To date, only one study has directly compared spitting, aspiration, and Salivette-based collection using untargeted LC–MS, reporting comparable results for spitting and aspiration but poor quantitative performance for Salivette [31]. However, chewed cotton swabs were used in that study, which likely introduced both sorbent-related and stimulation-related variability. In contrast, our ex vivo design minimized such confounding factors and allowed for a direct assessment of swab–matrix interactions. Consistent with previous reports stating that cotton swabs are known to sorb selected analytes [29], our results indicate that synthetic swabs have a substantially lower effect.

Nevertheless, swab-based sampling may still influence saliva composition depending on swab placement relative to salivary gland outlets. Consequently, future salivary metabolomics studies may benefit from microsampling strategies that selectively target individual salivary glands. It is well established that the biochemical composition of saliva depends on the salivary gland from which it originates. Gland-specific, targeted probe placement could therefore reduce analytical variability arising from inconsistent swab positioning within the oral cavity as well as from interindividual differences in salivary flow rates across glands, which ultimately translate into variability in overall salivary composition. Metabolic profiling of whole, parotid, and submandibular/sublingual saliva using standard collection methods has already been performed [32], demonstrating significant differences between these saliva types. However, it remains unclear which of these represents the most informative source of metabolic biomarkers for systemic disease diagnostics and monitoring.

In our study, both stimulated and resting saliva were analyzed. Most salivary metabolomics studies focus on a single saliva type, using either stimulated [33,34] or resting saliva [35,36]. Our results, in line with previous reports, demonstrate substantial differences in both metabolite identity and abundance between these two matrices. Specifically, undecenedioic acid exhibited a 14-fold higher abundance in resting saliva compared with stimulated saliva, whereas hexadecenoic acid showed a 10-fold higher abundance in stimulated saliva. Similarly, Figueira et al. reported a three-fold higher acetate concentration and a three-fold lower lactate concentration in resting saliva relative to stimulated saliva and identified metabolites detected exclusively in resting samples [37].

These pronounced differences are likely driven, at least in part, by the increased contribution of parotid secretion during chewing stimulation, as well as dilution effects associated with elevated salivary flow, which lead to greater saliva hydration and reduced metabolite concentrations [37]. Collectively, these findings indicate that stimulated and resting saliva represent distinct and complementary biological matrices. Therefore, the parallel analysis of both saliva types should be considered a methodological standard in salivary metabolomics to maximize metabolite coverage and the clinical relevance of biomarker discovery.

Salivary metabolomics represents a rapidly expanding research field due to the non-invasive nature of saliva collection and its potential to reflect systemic physiological states and pathological conditions. Continuous advances in analytical methodologies, together with increasing sensitivity and accuracy, will further facilitate the detection and quantification of a broad spectrum of metabolites [38]. However, metabolite coverage, abundance, and the clinical relevance of the generated data are strongly influenced by multiple pre-analytical and analytical factors. Therefore, following biomarker selection using untargeted approaches, careful optimization and standardization of extraction and analytical workflows are essential to enable reliable quantification of clinically relevant metabolites and to support targeted, biomarker-driven applications in salivary diagnostics.

This study has some limitations. Although the study group was relatively large com-pared to other methodological optimization studies, it should be noted that certain factors, such as the composition of resting and stimulated saliva, may vary depending on the individual-specific variables, including oral health status and other physiological conditions. Additionally, all metabolites were analyzed using a single LC-MS/MS platform, and some of the observed differences may therefore reflect platform-specific effects, such as matrix-dependent ionization variability, including suppression or enhancement. Future studies including larger and more diverse cohort as well as complementary analytical platforms are needed to confirm the robustness of these findings and assess their broader applicability, particularly with respect to the impact of saliva stimulation on metabolite coverage. 

## 4. Materials and Methods

### 4.1. Saliva Collection

Whole saliva was collected from five volunteers, after overnight fasting, in the morning to minimize circadian effects. Resting saliva was collected via passive drooling for approximately 5 min, while stimulated saliva was obtained following 1 min of gentle chewing, followed by centrifugation at 1000× *g* for 2 min at 4 °C. Participants refrained from eating, drinking, and orofacial movements before sampling to minimize variability. All saliva samples were stored at −80 °C until further analysis.

### 4.2. Saliva Sample Preparation for Metabolomics Analysis

Metabolites were extracted using different extraction protocols designed for urine and plasma [25], comprising distinct solvent systems and predefined saliva-to-solvent ratios, rather than solvent composition alone. For acetonitrile (ACN) and acetonitrile/methanol (ACN:MeOH (1:1, *v*/*v*)) extraction, 100 µL of saliva was mixed with 200 µL of extraction solvent (1:2 saliva-to-extraction solvent ratio, *v*/*v*) containing internal standards (imipramine, indoxyl sulfate-d4, ADME-d6, and lysine-d9; 1 µg/mL each (Toronto Research Chemicals, Toronto, ON, Canada)). Samples were mixed, incubated on ice for 10 min, and centrifuged at 10,000× *g* for 10 min at 4 °C, after which the supernatants were transferred to chromatographic vials. For isopropanol (IPA) extraction (Merck, Darmstadt, Germany), the procedure was the same as described above; however, only non-polar internal standards were used (imipramine and indoxyl sulfate-d4; 1 µg/mL each). Following centrifugation, sample supernatants were collected, evaporated under nitrogen, reconstituted in water/ACN (1:3, *v*/*v*), centrifuged (10,000× *g* for 5 min), and transferred to chromatographic vials.

Methyl tert-butyl ether (MTBE)-based extraction used a solvent ratio of 2.6:2:2.4 MTBE:MeOH:H_2_O (*v*/*v*/*v*), with a final saliva/extraction solvent ratio of 1:21.4 (*v*/*v*). Saliva aliquots were combined with ice-cold methanol containing internal standards (imipramine and indoxyl sulfate-d4, 1 µg/mL each, (Toronto Research Chemicals, Toronto, ON, Canada)) and ultrapure water (resistivity 18.2 MΩ·cm at 25 °C, obtained from a Milli-Q purification system (Merck Millipore, Burlington, MA, USA)), vortexed for 1 min, followed by the addition of MTBE and water. After additional mixing for 1 min, samples were incubated on ice for 10 min and centrifuged at 10,000× *g* for 10 min at 4 °C. An aliquot corresponding to 70% of the organic phase was collected, evaporated under a nitrogen stream, reconstituted in water–ACN (1:3, *v*/*v*) to match the saliva dilution used in the other extraction methods, centrifuged at 10,000× *g* for 5 min, and transferred to chromatographic vials.

Quality control (QC) samples were prepared by pooling equal aliquots of all saliva samples and were used to monitor the repeatability of the extraction procedure and the stability of the LC–MS system. Separate QC samples were prepared independently for each extraction method and processed in the same way as the study samples. QC samples were injected periodically (every 5 samples).

Samples were analyzed within a single batch, in a randomized order to minimize potential batch effects. 

### 4.3. Selection of the Swab Type

Stimulated and resting saliva samples were incubated in test tubes under three conditions: with cotton swabs, with synthetic swabs (Sarstedt AG & Co. KG, Nümbrecht, Germany), and without swabs (control). For each condition, five independent samples were prepared (*n* = 5). An aliquot of 1 mL of saliva was added to each tube and incubated at room temperature for 2 min to simulate clinical sample collection. Subsequently, swabs were stored at 4 °C for 2 h to mimic sample storage and transport conditions. Following incubation, saliva was recovered by centrifugation of the swabs according to the manufacturer’s instructions and stored at −80 °C until further analysis.

### 4.4. Instrumental Analysis

Instrumental metabolomics analysis was carried out using an Ultimate 3000 high-performance liquid chromatography (HPLC) system with an autosampler, column thermostat, and mixer, coupled to a high-resolution Orbitrap Focus mass spectrometer (Thermo Fisher Scientific, Waltham, MA, USA). Chromatographic separation was performed on four columns: SeQuant ZIC-HILIC (100 mm × 2.1 mm, 5 μm) (Merck, Darmstadt, Germany), Accucore Amide HILIC (100 mm × 4.6 mm, 2.6 μm) (Thermo Fisher Scientific, Waltham, MA, USA), Kinetex PFP (100 mm × 4.6 mm, 5 μm), and Kinetex C18 (100 mm × 4.6 mm, 2.6 μm) (Phenomenex, Torrance, CA, USA). The mobile phases consisted of (A) HPLC-grade water with 0.1% formic acid and (B) acetonitrile (Merck, Darmstadt, Germany) with 0.1% formic acid (Merck, Darmstadt, Germany). The column temperatures were maintained at 40 °C, and the flow rate was 0.3 mL/min. The injection volumes were 10 µL for C18 and PFP columns and 5 µL for HILIC columns. The gradient on Kinetex PFP and C18 was as follows: 20% B at 0 min, 20% B at 2 min, 90% B at 20 min, 90% B at 30 min, 20% B at 31 min and 20% B at 35 min. The gradient on HILIC columns was as follows: 90% B at 0 min, 90% B at 2.5 min, 50% B at 18 min, 50% B at 28 min, and 90% B at 34 min. Electrospray ionization (ESI) was performed in both positive and negative modes. Mass spectra were recorded in the m/z range of 75–1100 with a resolution of 70,000. Fragmentation spectra were acquired using collision energies of 20, 40 and 60 V (resolution 17,500). Standard mass spectrometric conditions in all experiments were as follows: spray voltage: 3.5 kV; sheath gas pressure: 60 arb; aux gas pressure: 20 arb; sweep gas pressure: 0 arb; heated capillary temperature: 320 °C; loop count: 3; isolation window: m/z 1.0; and dynamic exclusion: 6.0 s. For all full-scan measurements, lock-mass ions from ambient air (m/z 445.1200 and 291.2842) were used as internal calibrants.

### 4.5. Statistical Analysis

The metabolomic data were analyzed with Compound Discoverer 3.3 (Thermo Fisher Scientific, Waltham, MA, USA) (peak detection and annotations of metabolites) and MetaboAnalyst 6.0 (statistical analyses and data visualization). Peak areas of internal standards were used to correct for extraction and instrument variability. The data were log-transformed (base 10). Principal component analysis (PCA) was applied to evaluate the quality of the obtained data by evaluating clustering of the quality control samples. Furthermore, PCA, as an unsupervised method, was also used in data exploration. The heatmap obtained with row-standardized features was used to visualize patterns in changes in metabolite levels across samples. Euclidean distance was used as a measure of similarity, whereas Ward’s method was used for clustering. For comparisons between two groups, a t-test was applied (*p*-value cutoff of 0.05 for statistical significance), whereas analysis of variance (ANOVA) was used for comparisons involving more than two groups (*p*-value cutoff of 0.05 for statistical significance). The assumptions of the statistical tests were verified using Levene’s test and the Shapiro–Wilk test.

## 5. Conclusions

Saliva and swab type, extraction protocol, and the choice of chromatographic column all strongly influence the metabolome profiles obtained by LC–MS/MS analysis of saliva. In this study, we established an optimized workflow combining complementary C18 and ZIC-HILIC separations with a rapid and universal acetonitrile/methanol (ACN:MeOH (1:1, *v*/*v*)) extraction protocol compatible with both chromatographic modes. The clear metabolic differences between resting and stimulated saliva demonstrate that saliva type is a critical pre-analytical factor that can substantially influence the obtained results. The type of saliva should therefore be selected based on the specific diagnostic goal, and strict standardization is essential. Only one saliva type should be collected per experiment, with participants carefully instructed and monitored to follow the sampling protocol. For standardized sample collection, synthetic swabs are preferred over cotton swabs due to their lower impact on metabolite abundances. Given the substantial effect of both pre-analytical and analytical factors on metabolomic results, strict control and standardization are essential to ensure reproducibility, inter-study comparability, and reliable biological interpretation in salivary metabolomics.

## Data Availability

The original contributions presented in this study are included in the article/Supplementary Materials. Further inquiries can be directed to the corresponding author.

## Supplementary Materials

The following supporting information can be downloaded at https://www.mdpi.com/article/10.3390/ijms27083345/s1.

## References

[1] Marques L., Costa B., Pereira M., Silva A., Santos J., Saldanha L., Silva I., Magalhães P., Schmidt S., Vale N. (2024). Advancing Precision Medicine: A Review of Innovative In Silico Approaches for Drug Development, Clinical Pharmacology and Personalized Healthcare. Pharmaceutics.

[2] Surdu A., Foia L.G., Luchian I., Trifan D., Tatarciuc M.S., Scutariu M.M., Ciupilan C., Budala D.G. (2025). Saliva as a Diagnostic Tool for Systemic Diseases-A Narrative Review. Medicina.

[3] Zhao X., Chen X., Zhou Z., Zheng J., Lin Y., Zheng Y., Xu R., Hu S., Cui L. (2025). Advancements and Challenges in Salivary Metabolomics for Early Detection and Monitoring of Systemic Diseases. MedComm (2020).

[4] Dame Z.T., Aziat F., Mandal R., Krishnamurthy R. (2015). The human saliva metabolome. Metabolomics.

[5] Kim Y.J., Kim J.H., Hong A.R., Park K.S., Kim S.W., Shin C.S., Kim S.Y. (2020). Stimulated Salivary Cortisol as a Noninvasive Diagnostic Tool for Adrenal Insufficiency. Endocrinol. Metab..

[6] Klichowska-Palonka M., Pac-Kożuchowska E. (2009). Wykorzystanie badania śliny w diagnostyce endokrynologicznej. Endokrynol. Pediatryczna.

[7] Mandrell B.N., Avent Y., Walker B., Loew M., Tynes B.L., Crabtree V.M. (2018). In-Home Salivary Melatonin Collection: Methodology for Children and Adolescents. Dev. Psychobiol..

[8] Resztak M., Czyrski A., Sobiak J. (2025). Saliva as a matrix for therapeutic drug monitoring and disease biomarkers in children and adolescents. Pharmacol. Rep..

[9] Hyvärinen E., Savolainen M., Mikkonen J.J.W., Kullaa A.M. (2021). Salivary Metabolomics for Diagnosis and Monitoring Diseases: Challenges and Possibilities. Metabolites.

[10] Boudah S., Olivier M.-F., Aros-Calt S., Oliveira L., Fenaille F., Tabet J.-C., Junot C. (2014). Annotation of the human serum metabolome by coupling three liquid chromatography methods to high-resolution mass spectrometry. J. Chromatogr. B Anal. Technol. Biomed. Life Sci..

[11] Lepoittevin M., Blancart-Remaury Q., Kerforne T., Pellerin L., Hauet T., Thuillier R. (2023). Comparison between 5 extractions methods in either plasma or serum to determine the optimal extraction and matrix combination for human metabolomics. Cell. Mol. Biol. Lett..

[12] DeFelice B.C., Fiehn O., Belafsky P., Ditterich C., Moore M., Abouyared M., Beliveau A.M., Farwell D.G., Bewley A.F., Clayton S.M. (2022). Polyamine Metabolites as Biomarkers in Head and Neck Cancer Biofluids. Diagnostics.

[13] Li Z., Sarnat J.A., Liu K.H., Hood R.B., Chang C.-J., Hu X., Tran V., Greenwald R., Chang H.H., Russell A. (2022). Evaluation of the Use of Saliva Metabolome as a Surrogate of Blood Metabolome in Assessing Internal Exposures to Traffic- Related Air Pollution. Environ. Sci. Technol..

[14] Okuma N., Saita M., Hoshi N., Soga T., Tomita M., Sugimoto M., Kimoto K. (2017). Effect of masticatory stimulation on the quantity and quality of saliva and the salivary metabolomic profile. PLoS ONE.

[15] De Araújo C.S., Da Silva A.C.L., Freitas-Fernandes L., Maia L.C., Da Silva Fidalgo T., Valente A.P. (2024). Untargeted stimulated and unstimulated salivary metabolomics and saliva flow rate in children. Clin. Oral Investig..

[16] Foratori-Júnior G., Guennec A.L., Fidalgo T., Jarvis J., Mosquim V., Buzalaf M., Carpenter G., Sales-Peres S. (2023). Comparison of the Metabolic Profile between Unstimulated and Stimulated Saliva Samples from Pregnant Women with/without Obesity and Periodontitis. J. Pers. Med..

[17] Nam M., Jo S.r., Park J.H., Kim M.-S. (2023). Evaluation of critical factors in the preparation of saliva sample from healthy subjects for metabolomics. J. Pharm. Biomed. Anal..

[18] Fang L., Zhai Q., Zhang H., Ji P., Chen C., Zhang H. (2024). Comparisons of different extraction methods and solvents for saliva samples. Metabolomics.

[19] Chen X., Chen Y., Feng M., Huang X., Li C., Han F., Zhang Q., Gao X. (2022). Altered Salivary Microbiota in Patients with Obstructive Sleep Apnea Comorbid Hypertension. Nat. Sci. Sleep.

[20] Tanaka Y., Yamashita R., Kawashima J., Mori H., Kurokawa K., Fukuda S., Gotoh Y., Nakamura K., Hayashi T., Kasahara Y. (2022). Omics profiles of fecal and oral microbiota change in irritable bowel syndrome patients with diarrhea and symptom exacerbation. J. Gastroenterol..

[21] Mahalingam S.S., Jayaraman S., Bhaskaran N., Schneider E., Faddoul F., Silva A.P.d., Lederman M.M., Asaad R., Adkins-Travis K., Shriver L.P. (2023). Polyamine metabolism impacts T cell dysfunction in the oral mucosa of people living with HIV. Nat. Commun..

[22] Kim J., An S., Kim Y., Yoon D.-W., Son S.A., Park J.-W., Jhe W., Park C.-S., Shin H.-W. (2023). Surface Active Salivary Metabolites Indicate Oxidative Stress and Inflammation in Obstructive Sleep Apnea. Allergy Asthma Immunol. Res..

[23] Liu S., Zhang S., Chen H., Zhou P., Yang T., Lv J., Li H., Wang Y. (2023). Changes in the salivary metabolome in patients with chronic erosive gastritis. BMC Gastroenterol..

[24] Martias C., Baroukh N., Mavel S., Blasco H., Lefèvre A., Roch L., Montigny F., Gatien J., Schibler L., Dufour-Rainfray D. (2021). Optimization of Sample Preparation for Metabolomics Exploration of Urine, Feces, Blood and Saliva in Humans Using Combined NMR and UHPLC-HRMS Platforms. Molecules.

[25] Southam A.D., Haglington L.D., Najdekr L., Jankevics A., Weber R.J.M., Dunn W.B. (2020). Assessment of human plasma and urine sample preparation for reproducible and high-throughput UHPLC-MS clinical metabolic phenotyping. Analyst.

[26] Assad D.X., Mascarenhas E.C.P., Lima C.L.d., Toledo I.P.d., Chardin H., Combes A., Acevedo A.C., Guerra E.N.S. (2020). Salivary metabolites to detect patients with cancer: A systematic review. Int. J. Clin. Oncol..

[27] Schulte F., King O.D., Paster B.J., Moscicki A.-B., Yao T.-J., Dyke R.B.V., Shiboski C., Ryder M., Seage G., Hardt M. (2021). Salivary metabolite levels in perinatally HIV-infected youth with periodontal disease. Metabolomics.

[28] Teruya T., Goga H., Yanagida M. (2021). Human age-declined saliva metabolic markers determined by LC–MS. Sci. Rep..

[29] Campanella B., Legnaioli S., Onor M., Benedetti E., Bramanti E. (2023). The Role of the Preanalytical Step for Human Saliva Analysis via Vibrational Spectroscopy. Metabolites.

[30] He G., Dong T., Yang Z., Branstad A., Huang L., Jiang Z. (2022). Point-of-care COPD diagnostics: Biomarkers, sampling, paper-based analytical devices, and perspectives. Analyst.

[31] Bosman P., Pichon V., Acevedo A.C., Chardin H., Combes A. (2022). Development of analytical methods to study the salivary metabolome: Impact of the sampling. Anal. Bioanal. Chem..

[32] Meleti M., Quartieri E., Antonelli R., Pezzi M.E., Ghezzi B., Viani M.V., Setti G., Casali E., Ferrari E., Ciociola T. (2020). Metabolic Profiles of Whole, Parotid and Submandibular/Sublingual Saliva. Metabolites.

[33] Assad D.X., Acevedo A.C., Mascarenhas E.C.P., Normando A.G.C., Pichon V., Chardin H., Guerra E.N.S., Combes A. (2020). Using an Untargeted Metabolomics Approach to Identify Salivary Metabolites in Women with Breast Cancer. Metabolites.

[34] Herrala M., Mikkonen J.J.W., Pesonen P., Lappalainen R., Tjäderhane L., Niemelä R.K., Seitsalo H., Salo T., Myllymaa S., Kullaa A.M. (2020). Variability of salivary metabolite levels in patients with Sjögren’s syndrome. J. Oral Sci..

[35] Barnes V.M., Ciancio S.G., Shibly O., Xu T., Devizio W., Trivedi H.M., Guo L., Jönsson T.J. (2011). Metabolomics reveals Elevated Macromolecular Degradation in Periodontal Disease. J. Dent. Res..

[36] Wang Q., Gao P., Wang X., Duan Y. (2014). The early diagnosis and monitoring of squamous cell carcinoma via saliva metabolomics. Sci. Rep..

[37] Figueira J., Gouveia-Figueirab S., Öhmanc C., Holgersonc P.L., Nording M.L., Öhman A. (2017). Metabolite quantification by NMR and LC-MS/MS reveals differences between unstimulated, stimulated, and pure parotid saliva. J. Pharm. Biomed. Anal..

[38] Gardner A., Carpenter G., So P. (2020). Salivary Metabolomics: From Diagnostic Biomarker Discovery to Investigating Biological Function. Metabolites.

