# Supplementary material for: Impact of Selected Pre-Analytical and Analytical Factors on Untargeted Salivary Metabolomics
Source: Int J Mol Sci. 2026 Apr 8;27(8):3345. doi: 10.3390/ijms27083345 (PMC13116057; doi:10.3390/ijms27083345)
Supplement: Supplementary file 1 [file ijms-27-03345-s001.zip › ijms-4207735-supplementary.pdf]

*Supplementary data*

# Impact of Selected Pre-Analytical and Analytical Factors on Untargeted Salivary Metabolomics

**Sylwia Michorowska [1], Agnieszka Zięba, Dorota Olczak-Kowalczyk[2], Joanna Giebułtowicz *[1]**

[1]Department of Drug Chemistry, Pharmaceutical and Biomedical Analysis, Medical University of Warsaw, 02-091 Warszawa, Poland

[2] Department of Pediatric Dentistry, Medical University of Warsaw, 02-091 Warszawa, Poland

* Correspondence: joanna.giebultowicz@wum.edu.pl (JG ),

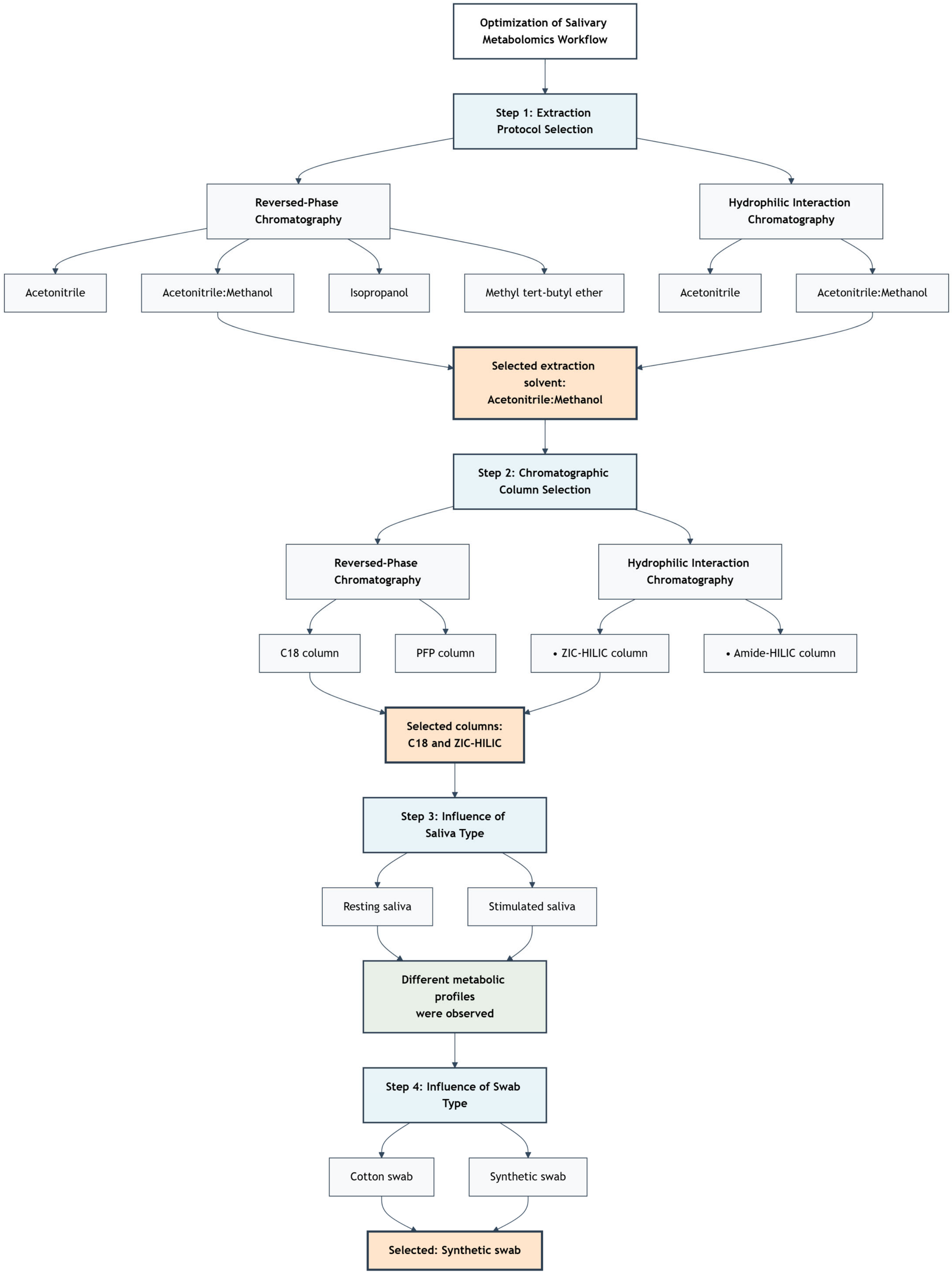


**Figure S1.** The workflow of the study

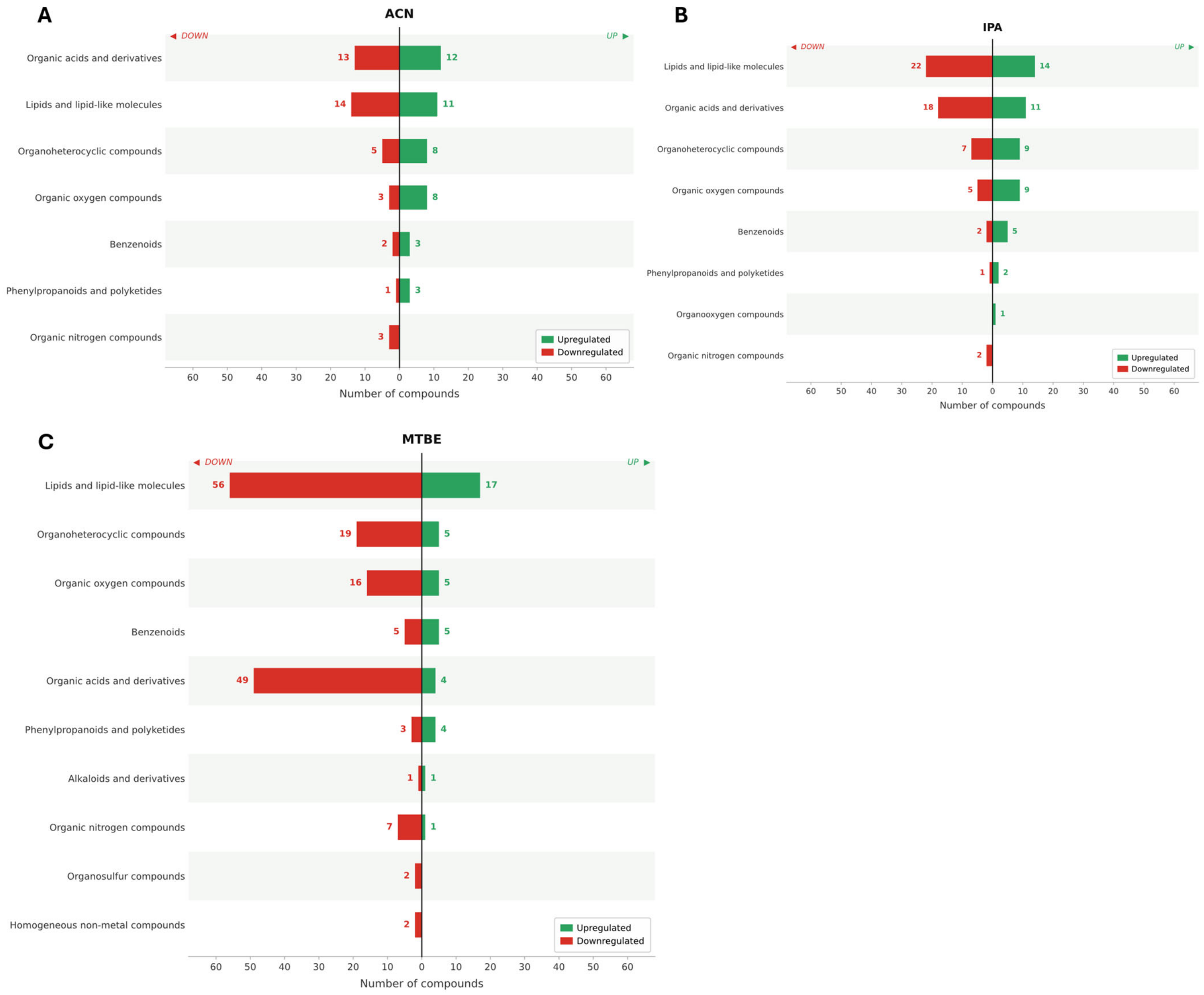


**Figure S2.** Distribution of metabolite superclasses detected in stimulated saliva using different extraction methods compared with the acetonitrile:methanol (ACN:MeOH (1:1, v/v)) extraction. Only those superclasses showing differences of at least two metabolites are presented. (A) ACN protocol; (B) IPA protocol; (C) MTBE protocol

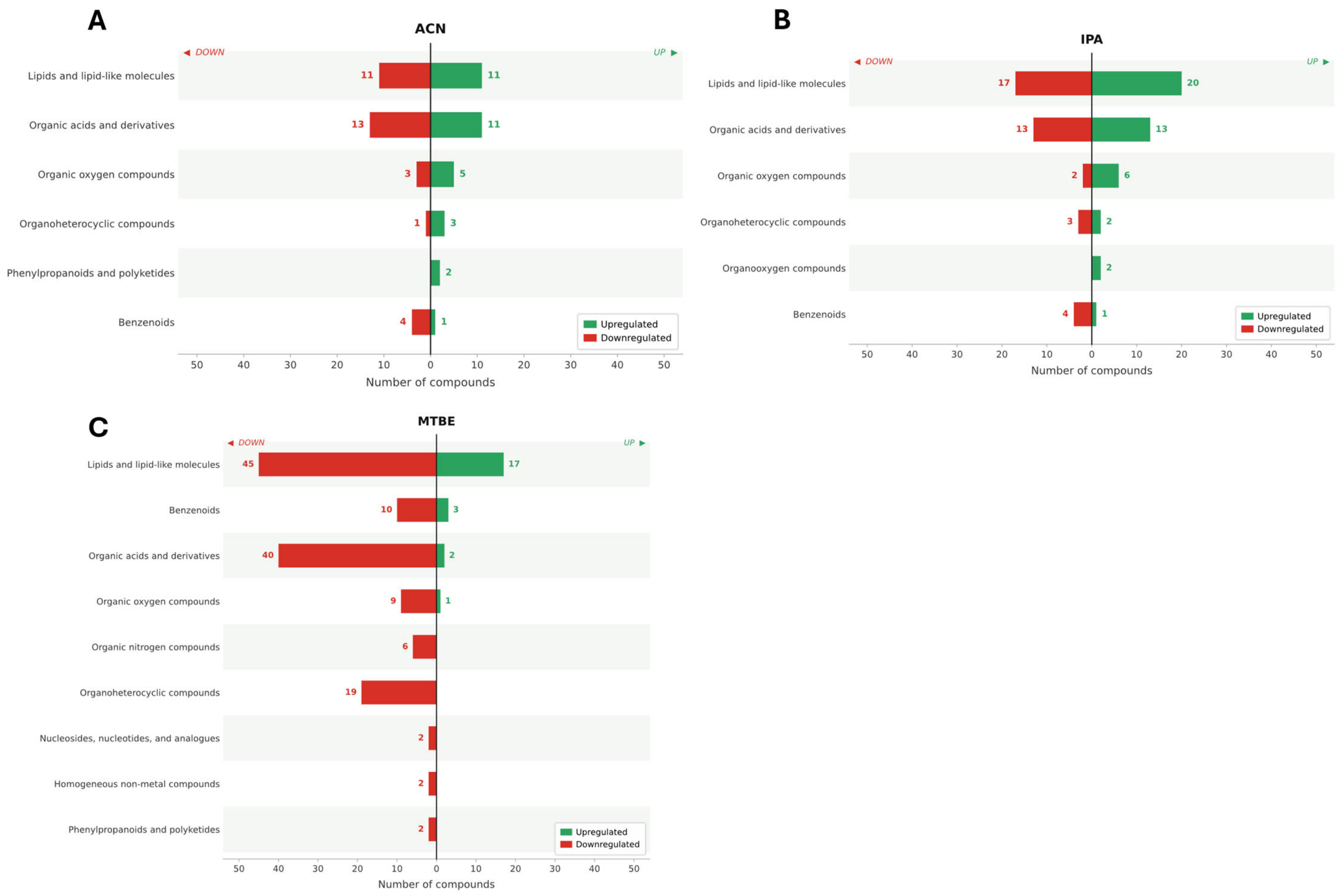


**Figure S3.** Distribution of metabolite superclasses detected in resting saliva using different extraction methods compared with the acetonitrile:methanol (ACN:MeOH (1:1, v/v)) extraction. Only those superclasses showing differences of at least two metabolites are presented. (A) ACN protocol; (B) IPA protocol; (C) MTBE protocol

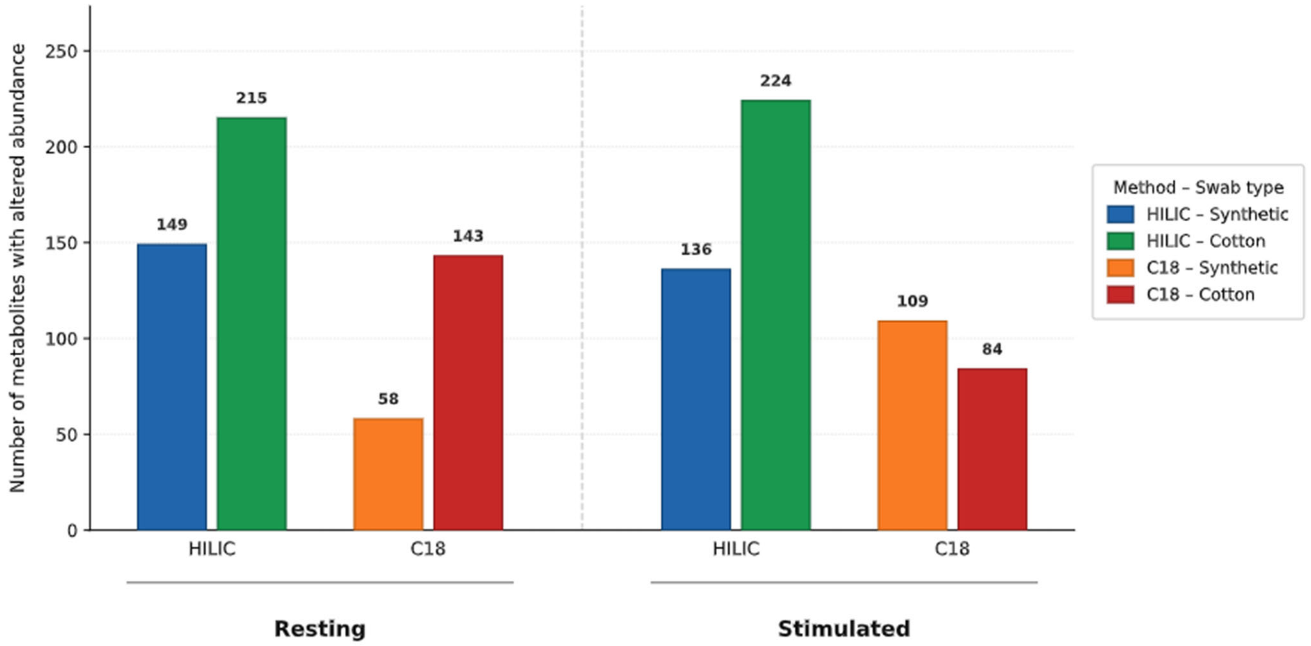


**Figure S4.** Effect of swab type on the number of metabolites with altered abundance after incubation with resting and stimulated saliva.
